# Management of Impella device fracture during direct aortic explantation

**DOI:** 10.1093/jscr/rjac637

**Published:** 2023-01-18

**Authors:** W Landon Jackson, Matthew J Kepford, Shane S Yamane, John F Carabello, Kryston R Boyer

**Affiliations:** Department of Surgery, Oklahoma State University Medical Center, Tulsa, OK 72211, USA; Department of Surgery, Oklahoma State University Medical Center, Tulsa, OK 72211, USA; Department of Surgery, Oklahoma State University Medical Center, Tulsa, OK 72211, USA; Department of Surgery, Oklahoma State University Medical Center, Tulsa, OK 72211, USA; Department of Surgery, Oklahoma State University Medical Center, Tulsa, OK 72211, USA

**Keywords:** Impella, aorta, bypass, cardiothoracic

## Abstract

The utilisation of mechanical circulatory support devices in patients undergoing high-risk coronary artery bypass continues to increase. The Impella is a ventricular assist device commonly used in the setting of post-cardiotomy cardiogenic shock. This device can be implanted directly into the ascending aorta during open cardiac surgery. Device fracture is a documented complication of the Impella; however, the management of device fracture during direct explantation from an aortic graft has not yet been described. We report a case of Impella device fracture during its removal from a prosthetic aortic graft and discuss the management of this complication.

## INTRODUCTION

The Impella device (Abiomed, Danvers, MA, USA) is commonly used for mechanical circulatory support in patients with post-cardiotomy cardiogenic shock [1, 2]. During cardiac surgery, the device can be directly implanted into the ascending aorta through a prosthetic graft, which is subsequently tunneled and brought out either to the chest wall or above the clavicle [3]. One described complication of the Impella is device fracture, although this has only been reported in cases where the device is implanted and explanted transcutaneously through the femoral approach [4, 5]. We describe a case of Impella device fracture during direct explantation from an aortic graft and discuss the management of this complication.

## CASE REPORT

A 47-year-old male presented with a chief complaint of chest pain and shortness of breath consistent with acute decompensated heart failure. He was diagnosed with non-ST-segment elevation myocardial infarction and underwent left heart catheterisation, which demonstrated multivessel coronary artery disease. Echocardiogram was significant for severe global hypokinesis with an ejection fraction of 14%. Additionally, the patient was suffering from acute renal failure secondary to cardiorenal syndrome. Prior to surgery, the patient temporarily required hemodialysis and inotropic support due to his decompensated heart failure. Ultimately, the decision was made to take the patient to surgery for high-risk aortocoronary bypass.

The patient underwent three-vessel coronary artery bypass without complication. After the bypasses were performed, a side-biting aortic clamp was applied approximately 7 cm from the aortic root. A 6-mm aortotomy was created. A 10-mm Dacron graft was then anastomosed to the aorta in an end-to-side fashion using a 5-0 prolene suture. The graft was tunneled to the right neck and brought out through a skin incision above the clavicle. The Impella 5.5 was inserted into the graft and advanced into the left ventricle under trans-esophageal echocardiogram (TEE) guidance. Excess graft material was trimmed and the securing device was advanced to the end of the graft. It was secured using heavy silk ties. The remaining graft was placed below the skin through the supraclavicular incision and the device was secured in place.

Post-operatively, the patient had a code blue event secondary to aspiration and respiratory arrest. This ultimately resulted in a non-perfusing ventricular arrhythmia. Advanced cardiopulmonary life support protocol was initiated and he briefly underwent chest compressions. He was successfully intubated and ventricular fibrillation was noted on the cardiac monitor. The patient was subsequently defibrillated with the return of spontaneous circulation. TEE was performed showing the Impella in the correct position. There was no evidence of damage to the device.

**Figure 1:**
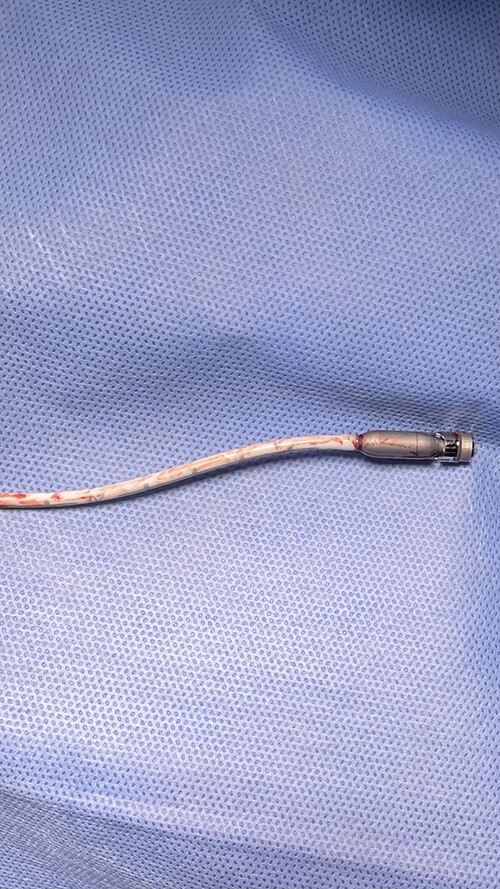
Fractured blood outlet portion and pump motor of Impella device.

**Figure 2:**
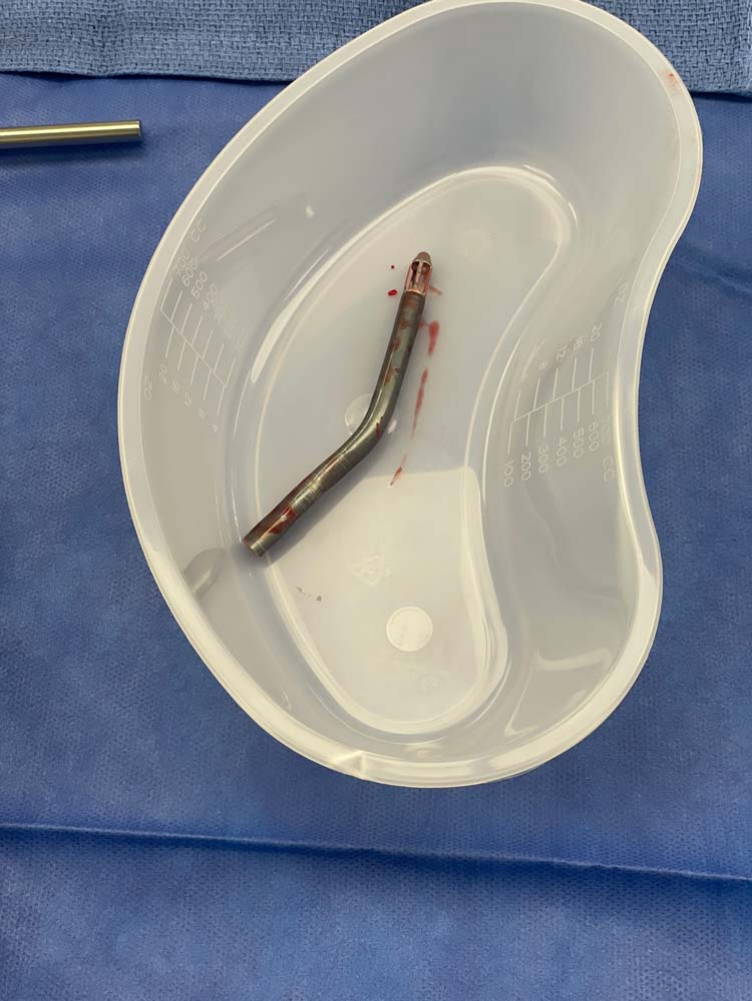
Fractured blood inlet portion of Impella device.

After this event, the patient’s clinical status improved and he was weaned from mechanical circulatory support. On post-operative day 7, the patient was taken back to the operating room for Impella device removal. The device and graft material were pulled from the incision to expose the excess vascular graft. The Impella was turned to the P1 setting and pulled back into the graft. As the device was being removed from the aorta, the plastic cannula broke apart from the outlet portion of the device (Fig. 1). The remaining portion of the device was within the graft itself (Fig. 2). A side-biting vascular clamp was applied to the externalised Dacron graft below the broken portion of the device to prevent migration back into the aorta. The graft was then rolled down to expose the outlet portion of the device and a Carmalt clamp was used to remove it from within the graft. The excess graft was then stapled closed using a linear cutting stapler. Skin was approximated over the residual graft material using a 2-0 nylon suture. The two pieces of the device were examined on the back table and TEE was performed to confirm complete explantation of the Impella device.

## DISCUSSION

The Impella device has demonstrated improved outcomes in the management of patients with post-cardiotomy cardiogenic shock [1, 2, 6]. The use of this device is generally considered to be safe; however, there have been multiple well-described complications associated with the Impella including damage to the aortic and mitral valves, hemolysis, vascular complications and bleeding [7–9]. Although device fracture has been reported in patients undergoing Impella explantation through the femoral approach, to our knowledge, device fracture during direct aortic explantation has not yet been described [4, 5].

Risks associated with Impella fracture during retrieval include massive hemorrhage and device embolisation. If device fracture occurs during direct aortic explantation, we recommend placing a heavy vascular clamp across the graft proximal to the device remnant to prevent retrograde migration into the aorta. Maintain manual control of the device until a heavy crushing clamp can be used to grasp across the device within the graft. After the device is grasped, excess graft can either be rolled down or cut in order to expose the device remnant such that it can be retrieved in its entirety. It is imperative to confirm complete retrieval of the Impella by examining the device after explantation. Evaluation with TEE is also recommended.

It is important to note that our patient underwent chest compressions while the Impella device was in place. We do not suspect damage to the Impella secondary to chest compressions as the device remained functional and no abnormalities were noted on echocardiogram; however, this cannot be entirely ruled out. Post-cardiotomy patients who develop cardiac arrest with an Impella should undergo defibrillation followed by bedside sternotomy if a blood pressure cannot be obtained. In this case, chest compressions were initiated by the intensive care unit (ICU) team. Upon arrival of the cardiothoracic service, the patient was successfully defibrillated and, therefore, did not require sternotomy.

Fortunately, our patient was in the operating room when this complication occurred. Although Impella explantation can be performed at the bedside in the ICU, we recommend a low threshold for performing explantation in the operating room where additional surgical instruments are readily available.

Finally, we recommend maintaining enough redundant graft to apply a clamp if needed. Although current recommendations include removing excess graft prior to device retrieval, this would not have allowed us to apply a clamp and ultimately grasp the device for removal.

## Data Availability

There is no data included in this manuscript.
